# What makes German manufacturing plants move locations?

**DOI:** 10.1007/s00168-023-01238-x

**Published:** 2023-09-08

**Authors:** Astrid Krenz

**Affiliations:** Department of Management and Economics, Center for Entrepreneurship, Innovation and Transformation (CEIT), Ruhr University Bochum, Universitaetsstrasse 150, 44801 Bochum, Germany

**Keywords:** D22, L22, R12, R30

## Abstract

In this paper, the relocation decisions of manufacturing plants across the NUTS-3 regions of the German economy are investigated. A relocation decision concerns whether a plant (an incumbent) moves its location from one region to another over a given time period or whether it remains in the same region. This decision is distinct from a location decision (of a start-up). To analyze the relocations of plants, the rich information of the official German regional statistics as well as the official German firm statistics that are maintained by the German Federal Statistical Office and the Statistical Offices of the Federal States is exploited for the first time. Both pull and push factors that influence relocation decisions are investigated. The results reveal that, in particular, regional road infrastructure and accessibility of regions as well as the quality of the available labor force positively affect the decision to relocate a plant in the German economy. A reduction of 10% in travel time by road to reach the three nearest agglomeration centers leads to an increase in relocation probability of about 9.5% on average. Policy implications involve the need for improvement of accessibility and infrastructure as well as incentives to support human capital in order to attract businesses to move to a region.

## Introduction

The German manufacturing industry is one of the most productive industrial sectors in the European Union and the world. However, in times of multiple crises, such as the recent pandemic, climate change, geopolitical tensions, and rising social inequality, German businesses face challenges. Several countries, such as the USA, have created large investment programs (such as the Inflation Reduction Act (IRA), the CHIPS and Science Act, or the Infrastructure Investment and Jobs Act), in order to subsidize the introduction of new firm activities. In this context, firm location decisions and regional economic or location policies have attracted renewed interest.

A recent endeavor of German politics has been to maintain and even raise the competitiveness of the country’s industrial sector, culminating with the release of the so-called National Industrial Strategy 2030. The aim of this strategy is to safeguard the German economy from rising competition from the USA and Asian countries. To achieve that broad goal, it is intended to promote new technologies—such as AI—and further innovation, to regain technological sovereignty by hindering the loss of technological know-how, and, importantly, to improve conditions for firm location, such as infrastructure, taxation and other costs, and labor market conditions (BMWI 2019; Lang 2022). Against this background, investigating where manufacturing firms choose to operate and why may deliver important policy implications for supporting regions’ attractiveness to business.

This paper focuses on the relocation decisions of manufacturing plants within the German economy. Relocation refers to a plant deciding to move its location from one area to another from one time period to another. Understanding what makes plants change their location can constitute a powerful lever to increase the attractiveness of an area in order to foster the establishment of firm activity and, consequently, the provision of employment. A higher number of people being in employment and thus, able to earn an income constitutes a crucial element of societal coherence and regional development.

Aside from the large enterprises, the German economy is known for its so-called Mittelstand, which comprises small and medium-sized businesses and family businesses (BDI 2021). These companies generate more than one-third of the total turnover of companies in Germany. Moreover, almost 60% of employees work in these businesses. They have often been rooted in rural regions and are highly productive in terms of research, development, and innovation. For the analyses of location choices, it will be important to address the heterogeneity of companies (for example in terms of size, i.e., the number of people employed).

The various German regions have different industrial structures and offer different employment opportunities. For example, in 2021, the number of people employed in the German manufacturing industry was more than 7.4 million, with around 107,000 employed in the manufacturing industry in Berlin, 1.4 million in North Rhine-Westphalia, 1.5 million in Bavaria, and 352,000 in Saxony (Destatis 2023). At the NUTS-3 level, the share of people employed in overall industry with regard to the working age population in 2020 was 8.36% in Berlin, 13.75% in Munich, 60.01% in Ingolstadt (where the automobile company Audi has its headquarters and main production facility), 31.03% in Bodenseekreis (in the South–West), and 10.15% in Vorpommern-Greifswald (the large district in the very North-East of the country) (BBSR 2023). As a consequence, the various German regions face different levels of risk in terms of economic decline, international crises, or other external shocks. For that reason, at the regional level, one more or one less plant can make a substantial difference.

Relocation decisions differ from location decisions by a fine degree of nuance. Investigating location decisions—which the bulk of the past literature has done—means disentangling the factors that lead a new plant/an entrant to decide in favor of one location. Investigating relocation decisions, however, involves finding the factors that attract a plant to locate itself in a new area, thus leaving its previous area, or stay in its current location. The relative lack of relocation decision studies compared to location decision studies is most likely due to insufficient data availability.

The aim of this paper is to investigate which factors drive the relocation decisions of manufacturing plants in the German economy. The German case is special due to its historical background: more than 30 years after German reunification, regional disparities, as well as differences in firm performance between regions in the East and in the West of Germany remain. It will be of particular interest to analyze how the relocation of businesses differs between these regions.

Regional economics literature has so far mainly studied the location decisions of new businesses (see, e.g., Armington and Zoltan 2002; Figueiredo et al. 2002; Rosenthal and Strange 2003; van Oort and Atzema 2004; Arauzo-Carod 2005; Feldman et al. 2005; Stam 2007; Glaeser and Kerr 2009; Glaeser et al. 2010; Audretsch et al. 2012; Fritsch and Wyrwich 2014; Glaeser et al. 2015; Artz et al. 2016; Fritsch and Wyrwich 2018; Dong 2020; Arauzo-Carod 2021; Krenz 2023). Further, a wide range of studies on new firm activity has focused on the localization of foreign direct investment (FDI) rather than domestic localization (see, e.g., Guimaraes et al. 2000; Procher 2011). However, there is less evidence available in the literature concerning relocation decisions. The present contribution is complementary to previous analyses that focus on location decisions of new firm activity, as well as studies that primarily analyze multiple alternative choices for location decisions at home or abroad (and FDI). The question here is whether or not (incumbent, i.e., existing and not new) plants decide to relocate, or, in other words, do plants “Stay or Move?”. Which characteristics of a region make it so attractive that plants decide to change their location (which involves leaving their present location and moving to another region)? Which factors of a region make plants want to stay? Finally: which policies are needed to attract plants to move to or stay in a region?

The categories of incumbent, that is, already existing, and new plants differ from one another. Consequently, one might assume that the reasons for relocation decisions made by existing plants will be different from the reasons for new plants’ location choices: Given that a location or relocation decision is undertaken on the grounds of incomplete information about the area or region, a business that already exists might benefit from its previous business experience, which in turn may influence its relocation decision (Pellenbarg et al. 2002).

The present study investigates for the first time the relocation decisions, rather than the location decisions, of manufacturing plants in Germany using the official firm statistics and comprehensive regional statistics held on the German economy. Relocation decisions are investigated across the NUTS-3 regions, that is, the district-free cities and districts (in German *kreisfreie Staedte und Kreise*), of Germany.^1^ Compared to the previous literature, the analysis makes use of a high-quality, large, newly constructed dataset on the German economy, with these data being derived from official regional statistics and data for the entire population of manufacturing firms in Germany. The analyses in this paper consider the relevance of pull (e.g., agglomeration economies and structural funding) and push factors (e.g., labor costs and taxes) for the relocation decisions of plants. The focus is thus on factors that make a plant choose either to move to a new location or to stay in its present location.^2^

For the analyses, probit regressions, as well as logit, Firth logit, and rare events logit modeling, were conducted. The results reveal that decisions to relocate are, on the one hand, driven by greater accessibility of a given region, as measured by travel times by road, and as such by the quality of road infrastructure. Specifically, a 10% decrease in travel time to the three nearest agglomeration centers leads to an increase in relocation probability of approximately 9.5% on average across all plants and 12.5% on average for small and medium-sized plants. This effect is relevant for small and medium-sized plants but not for large plants. A lower travel time makes small and medium-sized plants more likely to move toward another region. Furthermore, the quality of the workforce, as indicated by worker remuneration, positively affects the relocation choice. Again, this effect is relevant for small and medium-sized plants but not for large plants. Moreover, the potential to sell and find capacity in a market appears to be another driving factor in decisions to relocate, and this effect appears to be important for large plants.

The paper is structured as follows. Section 2 discusses the existing literature. In Sect. 3, the data are described. Section 4 presents the empirical analyses and interpretation of results. The final section offers conclusions.

## Literature review

There are many factors that can motivate a plant to undertake a relocation decision. The theoretical background to this issue emerges from the first writings about the location of economic activity by Marshall (1890) and the analytical framework of the New Economic Geography by Krugman (1991). As Manjon-Antolin and Arauzo-Carod (2011) describe, relocation decisions can be motivated by the same set of determinants as location decisions, the effect strength of the variables, however, can be expected to differ. Neoclassical, cost-related factors can be expected to determine relocation decisions, as a plant will make an optimal decision about a region to locate in and to maximize its profits. These factors comprise labor costs, market size, and agglomeration externalities. In addition to this, institutional factors can be expected to play a role: plants operate within regional systems and in an environment of regulations and laws, such that taxes, regional funding, and regional infrastructure are important factors for consideration (see Arauzo-Carod et al. 2010).

Among the existing studies on the relocation decisions of businesses, for the USA, Lee (2006) uses a probit model to investigate whether plants relocate and which factors drive that relocation. The author finds evidence that plants move out of regions in which industry is heavily concentrated to regions where it is not and describes these new regions as becoming new centers for industry. No evidence is found for the impact of taxes or human capital. Lee (2008) finds only weak effects of state development policies and incentive programs on the relocation of plants in the USA. Conroy et al. (2016) investigate relocations of firms, considering separately the effects of various factors on high-$R \& D$ and low-$R \& D$ firms. They find that the former tend to leave states that have a larger share of US manufacturing output in favor of states with a smaller share, are attracted to states that tax property more (which the authors interpret as pointing to the importance of amenities funded by property taxes), and make decisions relatively unaffected by transport infrastructure. The latter firms they find to be attracted to states with lower personal income taxes and states that spend more on higher education and transportation infrastructure. Conroy et al. (2017) find a link between higher wages and positive relocation decisions taken by firms in the US; they explain that this might be due to the decline in low-skilled manufacturing and the rise of high-tech manufacturing and reason that higher wages might indicate that highly-skilled labor is employed. Pan et al. (2020) analyze the relocation choices of firms in the USA and find that taxes and subsidies are important influential factors. Manjon-Antolin and Arauzo-Carod (2011) investigate the determinants of location and relocation decisions made by establishments across the constituent municipalities of Catalunya. Using a count data model, in their preferred specification, they find that relocation is motivated by urbanization and disurbanization economies, as well as human capital but not by infrastructure, location economies, or metropolitan effects. Holl (2004) investigates the relocation patterns of plants in Portugal, finding that greater market accessibility and provision of inter-regional motorways play an important role. Kronenberg (2013) finds that relocating firms in the Netherlands are generally attracted to densely populated municipalities with high wage levels. Weterings and Knoben (2013) find for the Netherlands that relocations within municipalities mainly depend on growth and the need for more space, while in terms of relocations across wider regions, firms stay in locations due to higher concentrations of other firms and greater urbanization and leave locations due to a higher share of innovative firms. The authors explain this as the result of congestion or competition. Knoben (2011) finds that a high level of agglomeration has a negative effect on the relocation of firms. Morkute and Koster (2018) investigate the importance of human capital for firm relocations in the Netherlands and find that human capital influences firms to some degree in deciding to stay but not in deciding to move. Investigating the relocation choices of firms in Poland, Rossi and Dej (2020) find that urbanization economies, availability of highly-skilled workers (indicated by higher pay), and amenities (public goods provision) appear to play an important role. Investigating the relocation choices of Norwegian firms, Nilsen et al. (2020) find that agglomeration, urbanization, and highly-skilled labor are variables that play an important role. Bodenmann and Axhausen (2012) find for Switzerland that relocation choices mainly depend on tax burdens and, to a lesser degree, on the accessibility of regions. Yi (2018) uses a nested logit modeling approach and finds that establishments in South Korea are attracted to relocate due to localization economies in earlier stages of the business life cycle, but not in later stages. Hong (2014) observes that Korean plants base relocation decisions on agglomeration economies. Brouwer et al. (2004) investigate relocation through a survey of 21 countries and focus on large firms with more than 200 employees. They find that larger and older firms are less likely to relocate, while firms serving larger markets are more likely to relocate.

In sum, the literature provides mixed evidence across a range of countries for the relevance of various factors that play a role in the relocation choices of firms. There has been evidence for the influence of urbanization and disurbanization economies (Manjon-Antolin and Arauzo-Carod 2011), (road) infrastructure (Holl 2004; Bodenmann and Axhausen 2012), and market size effects (Brouwer et al. 2004; Lee 2006; Conroy et al. 2016). There is less consensus as to the relevance of localization externalities (see, e.g., Manjon-Antolin and Arauzo-Carod 2011; Knoben 2011; Kronenberg 2013; Hong 2014; Yi 2018), human capital (Lee 2006; Manjon-Antolin and Arauzo-Carod 2011; Kronenberg 2013; Conroy et al. 2017; Morkute and Koster 2018; Nilsen et al. 2020; Rossi and Dej 2020), and taxes (Lee 2006, 2008; Bodenmann and Axhausen 2012; Conroy et al. 2016; Pan et al. 2020) for the relocation choices of firms. The impact of structural funding on firm relocation choices is not yet well explored: the previous literature has focused mainly on the macroeconomic impacts of such funding, such as regions’ GDP or regional employment, rather than firm relocation choices, which is likely to be due to lower data availability.

## Data

The dataset comprises regional information from the INKAR (Indicators and Maps for Spatial and Urban Development) database of the German Federal Institute for Research on Building, Urban Affairs and Spatial Development (BBSR) and from the Regional Database (GENESIS) of the German Federal Statistical Office. The regional data allow us to investigate plants’ decisions to relocate between the district-free cities and districts (*Landkreise und kreisfreie Staedte*, corresponding to the NUTS-3 level) of the German economy.

Generally, an analysis of location or relocation can be run at different regional levels, and it can provide important insights on any one of those, be it the country, state, county or community level. The choice of the regional level for the analyses in this paper was based on both the feasibility of computations given the large firm-level dataset and the availability of some of the explanatory variables at the level of NUTS-3 regions. The level of aggregation at the NUTS-3 level is meaningful because important policies are implemented at the level of districts and district-free cities. Several structural and regional funds are allocated—and have been for several decades—at the district level. This policy variable is one of the *x*-variables employed in the present regression framework. Moreover, labor markets are better captured at a more aggregated regional level than at the community level because many people commute to work across relatively substantial distances. This consideration also applies to the accessibility of regions and the infrastructure variable.

A range of variables was extracted from the data sources: Table 1 provides a detailed description of these variables. The size of the respective regional population and the number of manufacturing plants per capita in a region were used to model agglomeration economies (similar to Procher 2011). The bulk of the past literature employed population measures in estimating location or relocation choices (see, e.g., Guimaraes et al. 2000; Figueiredo et al. 2002; Manjon-Antolin and Arauzo-Carod 2011; Procher 2011; Kronenberg 2013). Many studies also applied specific measures for localization or urbanization economies. Guimaraes et al. (2000), Figueiredo et al. (2002), and Manjon-Antolin and Arauzo-Carod (2011), for example, used employment shares in either manufacturing or services to model agglomeration effects. Regional GDP per capita and GDP growth served as proxies for market size effects (see, e.g., Procher 2011). Labor costs, measured here as gross wages in manufacturing per employee, reflect, on the one hand, the costs of production that a plant has to pay and, on the other hand, the quality of the regional workforce (because highly-skilled workers are expected to be paid higher wages; see, e.g., Smith and Florida 1994). Tax revenues, travel times by road, and structural funding are institutional factors that model the provision of public goods, regional infrastructure, and regional policy effects.

**Table 1:** Description of variables

Variable	Description and measurement	Data	Mean	Std.Dev.	p(1)	p(99)	Obs.
Plant agglomeration	Number of plants in manufacturing, per population, logged measure	Regional Database GENESIS	1031.95	839.38	147	5504	43,278
Population	Number of population, logged measure	Regional Database GENESIS	327089.5	459299.4	47035	3375222	43,278
GDP	GDP per population, in thousands, in euros, logged measure	INKAR/BBSR based on *Arbeitskreis Volkswirtschaftliche Gesamtrechnung der Laender*, Eurostat Regio Database	31.4	10.78	17.7	73.5	43,278
GDP growth	GDP growth over past year, in percent	INKAR/BBSR based on *Arbeitskreis Volkswirtschaftliche Gesamtrechnung der Laender*, Eurostat Regio Database	1.93	2.77	− 5.2	9.6	43,278
Labor costs	Gross wages in manufacturing per employee in euros, logged measure	INKAR/BBSR based on *Monats- und Jahresbericht fuer Betriebe im Bereich Verarbeitendes Gewerbe, Bergbau und Gewinnung von Steinen und Erden*	3390.55	699.56	2074.7	5446.3	43,278
Taxes	Business tax revenue per population in euros, logged measure	INKAR/BBSR based on *Realsteuervergleich des Bundes und der Laender*	493.99	277.8	164.9	1828.6	43,278
Accessibility	Average travel time by car to the three nearest agglomeration centers, in minutes, logged measure	INKAR/BBSR based on *Erreichbarkeitsmodell by BBSR*	93.27	24.52	47	155	43,278
Structural funding	Structural regional funding of *Gemeinschaftsaufgabe Verbesserung der Regionalen Wirtschaftsstruktur* (GRW) for long-term infrastructure (over past ten years), per population, logged measure	INKAR/BBSR based on Database *Raumwirksame Mittel by BBSR*	130.85	274.41	1	1331.6	43,278

Taxes were measured as regional business tax revenue per capita. Some studies found a positive effect of taxes, potentially indicating that more public investment is undertaken from the higher tax income; other studies found no significant effect, at all (see, e.g., Carlton 1983; Artz et al. 2016). Taxation of firms in Germany involves several elements. One of these is a business tax on firms that varies between communities (the German *Gemeinden*). Information on this was available from the INKAR database and was used for the regression analyses. In INKAR, tax revenue is summed up at the level of the NUTS-3 region and expressed as per capita figure.^3^

Average travel time in minutes by road to the three nearest agglomeration centers^4^ is a variable that is directly recorded in the INKAR database. It measures the accessibility of a region and the quality of its road infrastructure. According to the precise BBSR definition, this variable captures the average travel time in minutes by car to the closest 3 of 36 agglomeration centers in Germany and the neighboring countries. The BBSR conducts regular accessibility analyses (through its so-called *Erreichbarkeitsmodell*, see BBSR 2019), showing, for example, that the most north-easterly German regions are peripheral in terms of accessibility, whereas the West German regions of the $\textit{Ruhrgebiet}$ are highly accessible. For its analyses, the BBSR uses georeferenced information on a set of 662,000 routes and 518,000 nodes for Germany and calculates travel times and distances using ArcGIS. For road traffic, the BBSR uses information on types of streets (*Autobahn* (highway), *Landstrasse* (country road), etc.), length of streets, velocity, and travel time. The shortest travel times and/or distances are computed and stored in time or distance matrices.

Regional structural funding is measured by the long-term spending on regional infrastructure over the past ten years by the German government’s ”Joint Task on Regional Economic Development” (*Gemeinschaftsaufgabe Verbesserung der Regionalen Wirtschaftsstruktur*, abbr. GRW) complemented by co-funding from the European Regional Development Fund (ERDF). This variable is directly available within the INKAR data. A zero amount of funding in the dataset was replaced with a value of one euro in order not to lose many observations within the regressions.^5^ GRW funding is a means to support investment in business and infrastructure for economic purposes in the German economy and to help even out regional disparities. This funding comes in addition to basic funding undertaken by the firm itself.

Data from the official firm statistics maintained by the German Federal Statistical Office and the Statistical Offices of the Federal States were merged. Because German law mandates that all enterprises and plants must report to the official statistics, this is the most comprehensive firm dataset that exists for Germany. Data were accessed for the years 2013 and 2012. Specifically, data for manufacturing plants (*Amtliche Firmendaten fuer Deutschland* (AFiD), *Industriebetriebe*, see RDC 2023) were used, covering plants that employ at least 20 employees. This cut-off is used in the AFiD data itself; its aim is to protect the sensitive data of micro-sized firms. Importantly, however, above that cut-off, the data cover the whole population of plants, rather than a random draw from it. Moreover, focusing on firms of this size means that any decision regarding relocation is less likely to be based on an individual decision by the owner or manager. This is important given that the dataset does not contain any manager- or owner-related micro-level variables. This could be a potential avenue for future research addressing individual-level influences on relocation decisions.

In the following section, separate analyses are presented for the groups of all plants, small and medium-sized plants, and large plants. Small and medium-sized plants are defined for the purpose of these analyses to be those with fewer than 250 employees and total annual revenues of up to 50 million euros, while large plants are those with at least 250 employees and total annual revenues of more than 50 million euros. This follows the definition of the European Commission. The data include information on each plant’s location. It is this information that allows us to model the relocation decisions of manufacturing plants and investigate how relocation choices are affected by a location’s characteristics. From the year 2012 to the year 2013, among the class of plants with at least 20 employees, 73 plants relocated ($Y=1$) and 43,205 plants stayed in their existing location (i.e., a NUTS-3 region; $Y=0$); of the sample of small and medium-sized plants, 68 relocated and 37,571 stayed in their existing location, and of the sample of large plants, 5 relocated and 5634 stayed.^6^ Regional reclassifications (merging or division of regions or changes in regional identifiers) were taken care of for the dataset.

## Empirical analysis

### Methodology

The basis of the analysis is the random utility model. In the context under consideration, a plant derives a utility from making a relocation choice given a set of alternatives. The plant will choose the alternative that promises the highest utility. In the context of the present paper, the plant’s relocation decision can be modeled using a discrete choice model.

Put simply, this paper analyzes how the relocation choice of a plant for time *t* (either $y = 1$ when having relocated to the destination region or $y = 0$ when having stayed in the origin region) is related to a set *x* of regional explanatory factors applicable in the location region. This framework is similar to the probit regression approach applied by Lee (2006).

More precisely, and as is explained in greater detail below, in formal terms, it is investigated how the characteristics of a district-free city or district in year $t-1$ to which a plant is relocating or in which the plant is remaining in year *t* are related to the relocation decision (to stay or to move to another region) of the plant from year $t-1$ to year *t*. The plant’s relocation choice is based on the characteristics of the region that the plant decides to relocate to (for the decision to move) as well as those of the plant’s origin region (for the decision to stay). Based on the data that could be accessed, relocation decisions are investigated for the year 2013.

As a first step, the official German firm data were programmed to be accessible for merging the NUTS-3 level information from the regional databases.^7^ Through this step, the explanatory variables for a plant’s chosen region in 2013 were obtained. In a second step, the data from 2013 were programmed to contain the related regional information from the previous year, 2012. The datasets and information obtained through these steps were then merged. In the next step, the dependent variable was constructed to be a dummy variable that is 1 when a plant changed its location (the district-free city or district, i.e., the NUTS-3 region) from 2012 to 2013 and 0 if it stayed in the same region from 2012 to 2013. The relocation decision was then estimated using a probit model that involved a binomial choice with a dichotomous dependent variable.

In the regressions, the lagged value of the explanatory regional variables (from time $t-1$ rather than *t*) was used to cope with endogeneity issues. This procedure is frequently applied in the location choice literature (see, for example, Kronenberg 2013). The lag structure aims to circumvent issues of reverse causality. Thus, it can be modeled that an investor bases their decision to relocate on the previous year’s regional characteristics.

More formally, the probability that a plant i chooses to relocate ($y=1$) is $P_{i1}=F(r_{i}, \beta )=\Phi (\beta r_{i})$, with r as a matrix of regional characteristics. Some of these characteristics are observed (which are the explanatory variables in the regression framework, for which coefficients will be estimated) and some remain unobserved. The plant derives a utility $U_{i}$ from making a relocation decision: $U_{i}=\beta r_{i} + \epsilon _{i}$. The plant chooses to relocate ($y=1$) when $U_{i} > 0$ and it decides to stay in the region ($y=0$) when $U_{i}<=0$. The error term is assumed to be standard normally distributed. The coefficients $\beta$ can be estimated and indicate the influence that the regional characteristics have in explaining the relocation decision of a plant in the German economy.

For robustness analyses, logit regressions, Firth logit regressions, and rare events logit regressions were also run. While the choice between probit and logit regressions is based on the underlying distributions of the error terms (either normal or logistic), Firth and rare events logit are designed to cope with rare events for the *y*-variable and small sample sizes. Firth logit is a penalized maximum likelihood estimation that is able to reduce bias in generalized linear models. Rare events logit provides bias correction as regards the underestimation of event probabilities.

### Results

#### Baseline results

Probit regression coefficients, as well as the average marginal effects (AME), are displayed in Table 2. Given that the whole population of firms and not a sample was used for the analysis, the results are assessed here with regard to both their statistical and economic significance. Robust standard errors were computed for the coefficients. For the AMEs, standard errors were computed based on the Delta-method; this method approximates the standard errors by the use of a first-order Taylor series approximation.

**Table 2:** Relocation choices, all plants

	(1)	(2)	(3)	(4)	(5)	(6)
	Probit coefficient	*Average marginal effect*	Probit coefficient	*Average marginal effect*	Probit coefficient	*Average marginal effect*
Plant agglomeration	− 0.025	− 0.0001	− 0.0263	− 0.0001	0.2492	0.0015
	(0.146)	(0.0008)	(0.1508)	(0.0009)	(0.166)	(0.001)
Population	− 0.0456	− 0.0002	− 0.0502	− 0.0003	− 0.0618	− 0.0004
	(0.0586)	(0.0003)	(0.0583)	(0.0003)	(0.0741)	(0.005)
GDP	− 0.4659*	− 0.0025*	− 0.5178**	− 0.003**	− 0.3726	− 0.0023
	(0.2502)	(0.0014)	(0.2577)	(0.0015)	(0.2763)	(0.0017)
GDP growth	− 0.0184	− 0.0001	− 0.0173	− 0.0001	− 0.0169	− 0.0001
	(0.0132)	(0.0001)	(0.0135)	(0.0001)	(0.0141)	(0.0001)
Labor costs	0.8668***	0.0046**	0.8796***	0.005**	1.0573***	0.0065***
	(0.3319)	(0.0018)	(0.332)	(0.002)	(0.346)	(0.0022)
Taxes	0.0699	0.0004	0.0853	0.0005	0.0274	0.0002
	(0.1349)	(0.0007)	(0.1435)	(0.0008)	(0.1467)	(0.0009)
Accessibility	− 0.3169*	− 0.0017*	− 0.3012*	− 0.0017*	− 0.5092***	− 0.0031**
	(0.1661)	(0.0009)	(0.1664)	(0.001)	(0.1979)	(0.0013)
Structural funding	− 0.0086	− 0.00005	− 0.0109	− 0.00006	0.0088	0.00005
	(0.0221)	(0.0001)	(0.0219)	(0.0001)	(0.0287)	(0.0002)
Industry fixed effects	No	No	Yes	Yes	Yes	Yes
Regional fixed effects	No	No	No	No	Yes	Yes
Wald $\chi ^{2}$	23.39		59.25		93.50	
Prob $>\chi ^{2}$	0.0029		0.0001		0.0000	
Log pseudolikelihood	− 524.289		− 504.247		− 494.145	
Number of plants	43,278		39,283		35,843	

This Table displays estimates for the relocation choice of German manufacturing plants. The dependent variable models the relocation decision $Y=1$ or the decision to stay $Y=0$ for each plant. Data are taken from the German Federal Statistical Office and the Statistical Offices of the Federal States, from the Regional Database GENESIS and from INKAR/BBSR. Robust standard errors are shown in parentheses for Columns (1), (3), and (5). Standard errors based on the Delta-method were computed and are shown in parentheses for Columns (2), (4), and (6). ***Denotes significance at the 1% level, **denotes significance at the 5% level, *denotes significance at the 10% level

The results of the regressions are supported by evidence found in previous literature and will be further explained below. The coefficients in Column (1) reveal that better regional road infrastructure, as measured by lower travel time by road to the three nearest agglomeration centers, and consequently, greater accessibility of a region, drives the relocation decisions of manufacturing plants in the German economy. Further statistically significant factors influencing relocation decisions are higher labor costs, a fact that indicates a highly educated workforce, and a lower regional GDP, which is indicative of the relevance of open, untapped market potential. These coefficients also attain a size that suggests them to be economically significant. The economic significance is further assessed below.

The results in Column (2) display the average marginal effects. Because most of the explanatory variables are in logs, the average marginal effects display an increase in the relocation probability due to a 1-unit change in the log of the explanatory variable. The interpretation of these effects would be rather unintuitive: what we would prefer to discuss is the percentage increase in the original variable. To obtain the increase in relocation probability due to a 10% change in the explanatory variable, one must take the average marginal effect from the table and multiply it by ln(1.1) (see, e.g., Cameron and Trivedi 2010).^8^

**Fig. 1:**
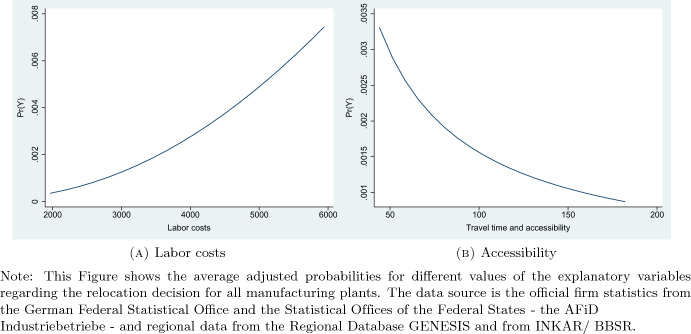
Predicted margins

Reducing travel time by 10% leads to an increase in the probability of relocating of 0.0162 percentage points (i.e., 0.000162 units). To obtain an impression of how large that effect is, we can determine the percentage increase in the relocation probability. The predicted probabilities differ across the values of the explanatory factors, which can be seen in Fig. 1. Let us take a look at the mean value for travel time, which is shown in the descriptive statistics in Table 1. The mean value for travel time is 93.2699 min. At that value, the predicted relocation probability is about 0.0017, as shown in Fig. 1, Panel (B). An increase of 0.000162 thus corresponds to a $\sim$ 9.5% increase in relocation probability, with the percentage increase computed as 0.000162/0.0017. A 10% decrease in travel time would be equivalent to a reduction of 9.3 min based on the mean value for travel time of 93.2699. The results find support in the study by Holl (2004), who shows that access to the inter-regional motorway network matters for the relocation choices of plants in Portugal.

A 10% increase in labor costs in a NUTS-3 region results in an increase in relocation probability of 0.044 percentage points. This corresponds to an increase in relocation probability of 23% (0.00044/0.0019) at the mean value of monthly labor costs (see Panel (A) of Fig. 1 and the descriptive statistics in Table 1). A 10% increase in labor costs would be an increase of 339.05 euros per capita per month. The positive impact of labor costs on firms’ location choices has previously been found in the literature and points to the relevance of a highly educated workforce, which is paid more highly (see, e.g., Smith and Florida 1994). Labor costs are able to better capture the quality of a region’s workforce than direct measures of student or graduate numbers given that those persons tend to be highly mobile and may not stay in the area which they earned their education in.

Moreover, the results in Table 2 show that a 10% increase in regional GDP per capita leads to a decrease in relocation probability of 0.0238 percentage points. This corresponds to a decrease in the relocation probability of 11.9% (0.000238/0.002) at the mean value of annual regional GDP per capita. A 10% increase in regional GDP would be an increase of 3140 euros per capita. This result could be interpreted in line with the evidence provided by Lee (2006), where plants are interpreted to leave old industry centers and move to regions with open, untapped market potential. However, another influence might also result from the cost of land. This is a factor that could not be directly controlled for in the regressions, given data availability. Besides, no statistically significant effects resulting from plant agglomeration, regional population, taxes, or structural funding were found.^9^ Columns (3) and (5) show the results when further industry (2-digit level) and regional (state-level) fixed effects were included in the regressions. The regressions show that the effects of labor costs and accessibility became stronger when further fixed effects were considered.

#### Analysis of firm heterogeneity

*Firm size*. The effects were further analyzed separately for small and medium-sized plants (those with fewer than 250 employees and total annual revenues of up to 50 million euros) and for the remaining group of large plants. Table 3 reveals that for small and medium-sized plants, statistically significant impacts result from greater accessibility and higher labor costs. Based on Column (1), the effect of road infrastructure is a 0.0238 percentage point increase in relocation probability given a 10% decrease in travel time by road to the three nearest agglomeration centers. At a mean value for travel time of 93.59, the relocation probability is about 0.0019.^10^ This corresponds to a $\sim$ 12.5% increase in the relocation probability. For a 10% increase in labor costs relocation probability increases by 0.0477 percentage points.

**Table 3:** Relocation choices, small and medium-sized plants

	(1)	(2)	(3)	(4)	(5)	(6)
	Probit coefficient	*Average marginal effect*	Probit coefficient	*Average marginal effect*	Probit coefficient	*Average marginal effect*
Plant agglomeration	− 0.0024	− 0.00001	− 0.0056	− 0.00003	0.2676	0.0018
	(0.1558)	(0.0009)	(0.1624)	(0.001)	(0.1757)	(0.012)
Population	− 0.0656	− 0.0004	− 0.0737	− 0.0005	− 0.0551	− 0.0004
	(0.0583)	(0.0003)	(0.0578)	(0.0004)	(0.0812)	(0.0005)
GDP	− 0.3494	− 0.002	− 0.3788	− 0.0023	− 0.1955	− 0.0013
	(0.2573)	(0.0015)	(0.2658)	(0.0016)	(0.2802)	(0.0019)
GDP growth	− 0.0189	− 0.0001	− 0.0184	− 0.0001	− 0.0184	− 0.0001
	(0.0131)	(0.0001)	(0.0134)	(0.0001)	(0.0143)	(0.0001)
Labor costs	0.8844**	0.005**	0.9027**	0.0055**	1.1036***	0.0074***
	(0.3569)	(0.0021)	(0.3578)	(0.0023)	(0.3836)	(0.0027)
Taxes	0.0014	0.0000	0.0117	0.00007	− 0.0722	− 0.0005
	(0.1302)	(0.0007)	(0.141)	(0.0009)	(0.1393)	(0.0009)
Accessibility	− 0.4431***	− 0.0025**	− 0.4364**	− 0.0027**	− 0.5719***	− 0.0038***
	(0.171)	(0.001)	(0.1706)	(0.0011)	(0.2077)	(0.0015)
Structural funding	− 0.0073	− 0.00004	− 0.008	− 0.00005	0.0099	0.00007
	(0.0235)	(0.0001)	(0.0234)	(0.0001)	(0.0302)	(0.0002)
Industry fixed effects	No	No	Yes	Yes	Yes	Yes
Regional fixed effects	No	No	No	No	Yes	Yes
Wald $\chi ^{2}$	25.41		54.42		90.88	
Prob > $\chi ^{2}$	0.0013		0.0002		0.0000	
Log pseudolikelihood	− 481.211		− 461.919		− 451.285	
Number of plants	37,639		33,749		30,169	

This Table displays estimates for the relocation choice of German manufacturing plants. The dependent variable models the relocation decision $Y=1$ or the decision to stay $Y=0$ for each plant. Data are taken from the German Federal Statistical Office and the Statistical Offices of the Federal States, from the Regional Database GENESIS and from INKAR/BBSR. Robust standard errors are shown in parentheses for Columns (1), (3), and (5). Standard errors based on the Delta-method were computed and are shown in parentheses for Columns (2), (4), and (6). ***Denotes significance at the 1% level, **denotes significance at the 5% level, *denotes significance at the 10% level

For large plants (those with at least 250 employees and total annual revenues of more than 50 million euros), statistically significant results are seen for road infrastructure, a higher tax base, and a lower regional GDP. Based on the results in Column (1) of Table 4, road infrastructure is not an important factor in the decision to relocate for large plants. A 10% increase in regional GDP leads to a 0.0562 percentage point decrease in relocation probability. With regard to business tax revenues, the results seem to indicate that large plants localize in regions that offer better public goods, which can be provided locally by a larger tax base (in line with evidence presented by Carlton 1983).

**Table 4:** Relocation choices, large plants

	(1)	(2)	(3)	(4)	(5)	(6)
	Probit coefficient	*Average marginal effect*	Probit coefficient	*Average marginal effect*	Probit coefficient	*Average marginal effect*
Plant agglomeration	− 0.2653**	− 0.0007	− 0.2482	− 0.0019	1.5999**	0.035*
	(0.1114)	(0.0005)	(0.2461)	(0.0021)	(0.6967)	(0.0188)
Population	0.3206	0.0009	0.641**	0.005	− 0.0618	− 0.0014
	(0.2749)	(0.0009)	(0.3055)	(0.0032)	(0.2723)	(0.006)
GDP	− 2.121***	− 0.0059*	− 3.3497***	− 0.026*	− 3.6999***	− 0.081*
	(0.824)	(0.0035)	(1.1014)	(0.0137)	(1.3214)	(0.0429)
GDP growth	− 0.0147	− 0.00004	− 0.0127	− 0.0001	0.083	0.0018
	(0.0778)	(0.0002)	(0.0877)	(0.0007)	(0.0915)	(0.0021)
Labor costs	0.4794	0.0013	− 0.0014	− 0.00001	1.4558	0.0319
	(0.9008)	(0.0026)	(1.2101)	(0.0094)	(1.2135)	(0.0289)
Taxes	1.1632*	0.0032	1.6712*	0.013	2.2741**	0.0498*
	(0.6016)	(0.0022)	(0.8563)	(0.0084)	(0.9708)	(0.0285)
Accessibility	1.5172***	0.0042*	2.8157***	0.0219**	1.9248*	0.0422
	(0.5679)	(0.0024)	(0.7074)	(0.0113)	(1.1404)	(0.0316)
Structural funding	− 0.0431	− 0.0001	− 0.1334**	− 0.001	− 0.252***	− 0.0055*
	(0.051)	(0.0001)	(0.0577)	(0.0006)	(0.0976)	(0.0033)
Industry fixed effects	No	No	Yes	Yes	Yes	Yes
Regional fixed effects	No	No	No	No	Yes	Yes
Wald $\chi ^{2}$	188.55		94.71		47.63	
Prob $> \chi ^{2}$	0.0000		0.0000		0.0000	
Log pseudolikelihood	− 35.953		− 25.205		− 18.331	
Number of plants	5639		1553		421	

This Table displays estimates for the relocation choice of German manufacturing plants. The dependent variable models the relocation decision $Y=1$ or the decision to stay $Y=0$ for each plant. Data are taken from the German Federal Statistical Office and the Statistical Offices of the Federal States, from the Regional Database GENESIS and from INKAR/BBSR. Robust standard errors are shown in parentheses for Columns (1), (3), and (5). Standard errors based on the Delta-method were computed and are shown in parentheses for Columns (2), (4), and (6). ***Denotes significance at the 1% level, **denotes significance at the 5% level, *denotes significance at the 10% level

*East–West differences.* As a next step, analyses were conducted for East and West German regions, separated by plant size group. The results in Table 5 show that greater accessibility as represented by road infrastructure positively influences relocation choices in East German regions. In particular, for small and medium-sized plants, the factors of accessibility, positive GDP growth, and a lower population play an important role in relocation choices in this wider region.^11^

**Table 5:** Relocation choices, All plants and small and medium-sized plants, East German regions

	(1)	(2)	(3)	(4)	(5)	(6)	(7)	(8)
	All plants				Small and medium-sized plants			
	Probit coefficient	*Average marginal effect*	Probit coefficient	*Average marginal effect*	Probit coefficient	*Average marginal effect*	Probit coefficient	*Average marginal effect*
Plant agglomeration	0.2619	0.0005	0.5148*	0.0032	0.4277	0.0007	0.644	0.0038
	(0.2677)	(0.0006)	(0.3059)	(0.0022)	(0.3708)	(0.0007)	(0.4273)	(0.0028)
Population	− 0.1055	− 0.0002	− 0.1001	− 0.0006	− 0.5857***	− 0.001*	− 0.6349***	− 0.0037
	(0.1607)	(0.0003)	(0.1627)	(0.001)	(0.1736)	(0.0006)	(0.2048)	(0.0023)
GDP	1.8648	0.0036	2.1895	0.0134	2.0131	0.0034	2.4087	0.0141
	(1.9072)	(0.004)	(1.9678)	(0.0132)	(2.0342)	(0.0038)	(2.0982)	(0.0137)
GDP growth	0.0129	0.00003	0.0148	0.0001	0.0263***	0.00004*	0.0314***	0.0002*
	(0.0081)	(0.00002)	(0.0101)	(0.00007)	(0.0066)	(0.00003)	(0.0105)	(0.0001)
Labor costs	− 0.0346	− 0.0001	0.138	0.0008	− 0.5725	− 0.001	− 0.7115	− 0.0042
	(0.9859)	(0.0019)	(0.9159)	(0.0056)	(0.87)	(0.0015)	(1.0049)	(0.0062)
Taxes	− 0.2459	− 0.0005	− 0.2927	− 0.0018	− 0.0331	− 0.00006	0.029	0.0002
	(0.7995)	(0.0016)	(0.8456)	(0.0052)	(1.0177)	(0.0018)	(1.0551)	(0.0062)
Accessibility	− 1.0141*	− 0.002	− 0.9365*	− 0.0057	− 2.2193***	− 0.0037*	− 2.2159***	− 0.013*
	(0.5983)	(0.0014)	(0.5556)	(0.004)	(0.7679)	(0.0023)	(0.7776)	(0.0074)
Structural funding	− 0.0855	− 0.0002	− 0.071	− 0.0004	− 0.1274	− 0.0002	− 0.1284	− 0.0008
	(0.0948)	(0.0002)	(0.0938)	(0.0006)	(0.0831)	(0.0002)	(0.0837)	(0.0006)
Industry fixed effects	No	No	Yes	Yes	No	No	Yes	Yes
Wald $\chi ^{2}$	430.57		179.13		393.09		127.24	
Prob $>\chi ^{2}$	0.00		0.0000		0.000		0.000	
Log pseudolikelihood	− 38.99		− 31.603		− 30.212		− 23.1998	
Number of plants	8533		2346		7728		1841	

This Table displays estimates for the relocation choice of German manufacturing plants. The dependent variable models the relocation decision $Y=1$ or the decision to stay $Y=0$ for each plant. Data are taken from the German Federal Statistical Office and the Statistical Offices of the Federal States, from the Regional Database GENESIS and from INKAR/BBSR. Robust standard errors are shown in parentheses for Columns (1), (3), (5), and (7). Standard errors based on the Delta-method were computed and are shown in parentheses for Columns (2), (4), (6), and (8). ***Denotes significance at the 1% level, **denotes significance at the 5% level, *denotes significance at the 10% level

For the West German regions, the results are shown for all plants in Table 6, for small and medium-sized plants in Table 7, and for large plants in Table 8. The results from Table 6 demonstrate that greater accessibility of regions, higher labor costs, and lower GDP play a role in relocation decisions across all plants. From Table 7, one can see that greater accessibility and higher labor costs explain the relocation choices of small and medium-sized plants in particular, whereas, from Table 8, it can be seen that a lower GDP and a higher tax base are drivers for the relocation decisions of large plants.

**Table 6:** Relocation choices, all plants, West German regions

	(1)	(2)	(3)	(4)	(5)	(6)
	Probit coefficient	*Average marginal effect*	Probit coefficient	*Average marginal effect*	Probit coefficient	*Average marginal effect*
Plant agglomeration	0.0060	0.00004	0.0049	0.00003	0.2247	0.0015
	(0.1550)	(0.0009)	(0.1606)	(0.0011)	(0.1769)	(0.0012)
Population	− 0.0237	− 0.0001	− 0.0289	− 0.0002	− 0.0346	− 0.0002
	(0.0727)	(0.0004)	(0.0714)	(0.0005)	(0.0757)	(0.0005)
GDP	− 0.5135**	− 0.0031**	− 0.5692**	− 0.0037**	− 0.4106	− 0.0027
	(0.2476)	(0.0016)	(0.2565)	(0.0017)	(0.281)	(0.0019)
GDP growth	− 0.0214	− 0.0001	− 0.0201	− 0.0001	− 0.0205	− 0.0001
	(0.0146)	(0.00009)	(0.0149)	(0.0001)	(0.0153)	(0.0001)
Labor costs	0.7974**	0.0049**	0.8143**	0.0053**	1.0273***	0.0067***
	(0.3717)	(0.0023)	(0.375)	(0.0025)	(0.3674)	(0.0025)
Taxes	0.0893	0.0005	0.1051	0.0007	0.035	0.0002
	(0.1361)	(0.0008)	(0.1453)	(0.001)	(0.1496)	(0.001)
Accessibility	− 0.2894*	− 0.0018*	− 0.268	− 0.0018	− 0.4352**	− 0.0029***
	(0.1693)	(0.0011)	(0.1688)	(0.0011)	(0.2014)	(0.0014)
Structural funding	0.0120	0.0001	0.0102	0.0001	0.0099	0.00006
	(0.0272)	(0.0002)	(0.0273)	(0.0002)	(0.0285)	(0.0002)
Industry fixed effects	No	No	Yes	Yes	Yes	Yes
Regional fixed effects	No	No	No	No	Yes	Yes
Wald $\chi ^{2}$	15.82		63.97		79.41	
Prob $> \chi ^{2}$	0.045		0.0000		0.0000	
Log pseudolikelihood	− 482.13		− 461.779		− 449.141	
Number of plants	34,745		31,487		30,771	

This Table displays estimates for the relocation choice of German manufacturing plants. The dependent variable models the relocation decision $Y=1$ or the decision to stay $Y=0$ for each plant. Data are taken from the German Federal Statistical Office and the Statistical Offices of the Federal States, from the Regional Database GENESIS and from INKAR/BBSR. Robust standard errors are shown in parentheses for Columns (1), (3), and (5). Standard errors based on the Delta-method were computed and are shown in parentheses for Columns (2), (4), and (6). ***Denotes significance at the 1% level, **denotes significance at the 5% level, *denotes significance at the 10% level

**Table 7:** Relocation choices, Small and medium-sized plants, West German regions

	(1)	(2)	(3)	(4)	(5)	(6)
	Probit coefficient	*Average marginal effect*	Probit coefficient	*Average marginal effect*	Probit coefficient	*Average marginal effect*
Plant agglomeration	0.0241	0.0002	0.0174	0.0001	0.2354	0.0017
	(0.1662)	(0.0011)	(0.172)	(0.0012)	(0.1857)	(0.0013)
Population	− 0.0073	− 0.00005	− 0.0145	− 0.0001	− 0.0253	− 0.0002
	(0.079)	(0.0005)	(0.0776)	(0.0006)	(0.083)	(0.0006)
GDP	− 0.392	− 0.0026	− 0.4291	− 0.0031	− 0.2344	− 0.0017
	(0.2543)	(0.0017)	(0.2636)	(0.0019)	(0.2845)	(0.002)
GDP growth	− 0.0223	− 0.0001	− 0.0217	− 0.0002	− 0.0225	− 0.0002
	(0.0146)	(0.0001)	(0.0149)	(0.0001)	(0.0156)	(0.0001)
Labor costs	0.8122**	0.0054**	0.8382**	0.006**	1.0676***	0.0076**
	(0.4008)	(0.0027)	(0.4052)	(0.003)	(0.4089)	(0.003)
Taxes	0.0053	0.00004	0.017	0.0001	− 0.0652	− 0.0005
	(0.1294)	(0.0009)	(0.1408)	(0.001)	(0.1414)	(0.001)
Accessibility	− 0.3894**	− 0.0026**	− 0.3753**	− 0.0026**	− 0.4919**	− 0.0035**
	(0.1759)	(0.0012)	(0.1747)	(0.0013)	(0.2116)	(0.0016)
Structural funding	0.0161	0.0001	0.0155	0.0001	0.0106	0.00008
	(0.0281)	(0.0002)	(0.0283)	(0.0002)	(0.0299)	(0.0002)
Industry fixed effects	No	No	Yes	Yes	Yes	Yes
Regional fixed effects	No	No	No	No	Yes	Yes
Wald $\chi ^{2}$	18.47		53.14		71.50	
Prob > $\chi ^{2}$	0.0180		0.0004		0.0000	
Log pseudolikelihood	− 446.244		− 427.751		− 415.523	
Number of plants	29,911		26,825		26,229	

This Table displays estimates for the relocation choice of German manufacturing plants. The dependent variable models the relocation decision $Y=1$ or the decision to stay $Y=0$ for each plant. Data are taken from the German Federal Statistical Office and the Statistical Offices of the Federal States, from the Regional Database GENESIS and from INKAR/BBSR. Robust standard errors are shown in parentheses for Columns (1), (3), and (5). Standard errors based on the Delta-method were computed and are shown in parentheses for Columns (2), (4), and (6). ***Denotes significance at the 1% level, **denotes significance at the 5% level, *denotes significance at the 10% level

**Table 8:** Relocation choices, large plants, West German regions

	(1)	(2)	(3)	(4)	(5)	(6)
	Probit coefficient	*Average marginal effect*	Probit coefficient	*Average marginal effect*	Probit coefficient	*Average marginal effect*
Plant agglomeration	− 0.0100	− 0.00002	0.4143	0.0026	1.9287**	0.035*
	(0.1068)	(0.0003)	(0.3573)	(0.0026)	(0.8545)	(0.0193)
Population	− 0.2970**	− 0.0007	− 0.1926	− 0.0012	− 0.1423	− 0.0026
	(0.1396)	(0.0005)	(0.3153)	(0.0021)	(0.3226)	(0.006)
GDP	− 2.8164***	− 0.007*	− 4.182***	− 0.0263*	− 3.9337***	− 0.0716*
	(0.9455)	(0.004)	(1.102)	(0.0139)	(1.491)	(0.0419)
GDP growth	− 0.0111	− 0.00003	0.0295	0.0002	0.1021	0.0019
	(0.0675)	(0.0002)	(0.0933)	(0.0006)	(0.0964)	(0.0019)
Labor costs	0.4798	0.0012	0.502	0.0032	1.3206	0.024
	(1.2761)	(0.0032)	(1.7584)	(0.0112)	(1.5398)	(0.0289)
Taxes	1.6324***	0.004*	2.2505***	0.0142*	2.4098**	0.0438
	(0.6358)	(0.0025)	(0.8353)	(0.0083)	(1.0321)	(0.0268)
Accessibility	1.1815**	0.0029	2.9348***	0.0185*	2.2894**	0.0417
	(0.5177)	(0.0018)	(0.6848)	(0.0097)	(1.1266)	(0.0288)
Structural funding	− 0.1189	− 0.0003	− 0.2731**	− 0.0017	− 0.361**	− 0.0066
	(0.0959)	(0.0003)	(0.1381)	(0.0012)	(0.1612)	(0.0043)
Industry fixed effects	No	No	Yes	Yes	Yes	Yes
Regional fixed effects	No	No	No	No	Yes	Yes
Wald $\chi ^{2}$	161.47		63.76		35.59	
Prob $>\chi ^{2}$	0.0000		0.000		0.0000	
Log pseudolikelihood	− 26.825		− 16.545		− 13.357	
Number of plants	4834		1289		367	

This Table displays estimates for the relocation choice of German manufacturing plants. The dependent variable models the relocation decision $Y=1$ or the decision to stay $Y=0$ for each plant. Data are taken from the German Federal Statistical Office and the Statistical Offices of the Federal States, from the Regional Database GENESIS and from INKAR/BBSR. Robust standard errors are shown in parentheses for Columns (1), (3), and (5). Standard errors based on the Delta-method were computed and are shown in parentheses for Columns (2), (4), and (6). ***Denotes significance at the 1% level, **denotes significance at the 5% level, *denotes significance at the 10% level

#### Further robustness analyses

*Methodological approaches.* Further robustness analyses were run. Table 9 displays the results from running Firth logit and rare events logit estimations (see King and Zheng 2001) and Table 10 displays results from logit estimations. In summary, the results support the evidence gained from running probit regressions. Greater accessibility of regions and higher labor costs appear to be a particularly influential factor in the relocation choices of small and medium-sized plants. For large plants, accessibility and labor costs do not seem to be important, while a lower regional GDP appears to be important. Importantly, looking at the marginal effects from logit estimations in Table 10, one can see that they strongly resemble those from probit regressions in Tables 2, 3, and 4. The results from Firth logit and rare events logit estimations in Table 9 show coefficients that resemble those from logit estimations in Table 10. We can conclude, therefore, that the effects in the found relationships are relevant and potentially somewhat larger than the figures yielded by conventional probit estimators. Importantly, the same significances as those seen in the benchmark probit regressions are also found by the Firth logit and rare events logit estimators.

**Table 9:** Robustness analyses, Firth logit and rare events logit

	(1)	(2)	(3)	(4)	(5)	(6)
	All plants		Small and medium-sized plants		Large plants	
	Firth logit coefficient	RE logit coefficient	Firth logit coefficient	RE logit coefficient	Firth logit coefficient	RE logit coefficient
Plant agglomeration	− 0.0619	− 0.0626	0.0042	0.0032	− 1.3809	− 1.4556***
	(0.4994)	(0.4582)	(0.5169)	(0.4874)	(1.6106)	(0.2099)
Population	− 0.1431	− 0.1435	− 0.2029	− 0.2035	0.6334	0.5763
	(0.1954)	(0.1868)	(0.2048)	(0.1839)	(0.6844)	(1.0414)
GDP	− 1.4616*	− 1.4644*	− 1.1205	− 1.124	− 4.8756**	− 4.7951**
	(0.7544)	(0.76)	(0.7504)	(0.7804)	(2.2631)	(2.359)
GDP growth	− 0.0591	− 0.0591	− 0.0599	− 0.06	− 0.027	− 0.0336
	(0.0437)	(0.0417)	(0.0448)	(0.0407)	(0.200)	(0.3007)
Labor costs	2.8907***	2.8893***	2.9598***	2.9583***	1.4374	1.772
	(1.0431)	(1.0244)	(1.0661)	(1.1084)	(3.0773)	(2.7422)
Taxes	0.1967	0.2001	− 0.008	− 0.0038	2.8182*	2.7324
	(0.5022)	(0.4156)	(0.4923)	(0.3912)	(1.5575)	(1.6985)
Accessibility	− 1.0244**	− 1.0241**	− 1.4028***	− 1.4024***	4.0285*	3.9267**
	(0.522)	(0.5152)	(0.5417)	(0.5253)	(2.1889)	(1.8705)
Structural funding	− 0.0203	− 0.0201	− 0.017	− 0.0167	− 0.1223	− 0.09
	(0.0793)	(0.0707)	(0.0823)	(0.075)	(0.2588)	(0.1967)
Wald $\chi ^{2}$	30.11		33.33		14.71	
Prob $> \chi ^{2}$	0.0002		0.0001		0.0651	
Log pseudolikelihood	− 510.698		− 467.827		− 33.559	
Number of plants	43,278		37,639		5639	

This Table displays estimates for the relocation choice of German manufacturing plants. The dependent variable models the relocation decision $Y=1$ or the decision to stay $Y=0$ for each plant. Data are taken from the German Federal Statistical Office and the Statistical Offices of the Federal States, from the Regional Database GENESIS and from INKAR/BBSR. Robust standard errors were computed and are shown in parentheses. ***Denotes significance at the 1% level, **denotes significance at the 5% level, *denotes significance at the 10% level

**Table 10:** Robustness analyses, Logit

	(1)	(2)	(3)	(4)	(5)	(6)
	All plants		Small and medium-sized plants		Large plants	
	Logit coefficient	*Average marginal effect*	Logit coefficient	*Average Average marginal effect*	Logit coefficient	*Average Average marginal effect*
Plant agglomeration	− 0.0325	− 0.0001	0.0313	0.00006	− 0.6653***	− 0.0006*
	(0.4583)	(0.0008)	(0.4875)	(0.0009)	(0.2102)	(0.0003)
Population	− 0.1286	− 0.0002	− 0.1884	− 0.0003	1.0142	0.0009
	(0.1869)	(0.0003)	(0.184)	(0.0003)	(1.0431)	(0.001)
GDP	− 1.5093**	− 0.0025*	− 1.1830	− 0.0021	− 5.8058**	− 0.0051*
	(0.7602)	(0.0013)	(0.7806)	(0.0014)	(2.3628)	(0.0031)
GDP growth	− 0.0592	− 0.0001	− 0.0602	− 0.0001	− 0.0265	− 0.00002
	(0.0417)	(0.0001)	(0.0408)	(0.0001)	(0.3012)	(0.0003)
Labor costs	2.8055***	0.0047***	2.8682***	0.0052**	0.9701	0.0009
	(1.0246)	(0.0018)	(1.1087)	(0.0021)	(2.7465)	(0.0025)
Taxes	0.2319	0.0004	0.0407	0.00007	3.1337*	0.0028
	(0.4157)	(0.0007)	(0.3913)	(0.0007)	(1.7012)	(0.0019)
Accessibility	− 1.0454**	− 0.0018**	− 1.4242***	− 0.0026***	4.4059**	0.0039
	(0.5153)	(0.0009)	(0.5254)	(0.001)	(1.8735)	(0.0024)
Structural funding	− 0.0266	− 0.00004	− 0.0238	− 0.00004	− 0.1584	− 0.0001
	(0.0707)	(0.0001)	(0.0750)	(0.0001)	(0.197)	(0.0002)
Wald $\chi ^{2}$	25.44		27.44		440.86	
Prob $> \chi ^{2}$	0.0013		0.0006		0.0000	
Log pseudolikelihood	− 523.954		− 480.783		− 36.355	
Number of plants	43,278		37,639		5639	

This Table displays estimates for the relocation choice of German manufacturing plants. The dependent variable models the relocation decision $Y=1$ or the decision to stay $Y=0$ for each plant. Data are taken from the German Federal Statistical Office and the Statistical Offices of the Federal States, from the Regional Database GENESIS and from INKAR/BBSR. Robust standard errors are shown in parentheses for Columns (1), (3), and (5). Standard errors based on the Delta-method were computed and are shown in parentheses for Columns (2), (4), and (6). ***Denotes significance at the 1% level, **denotes significance at the 5% level, *denotes significance at the 10% level

*Plant-level interactions.* Additional robustness checks were run incorporating the cross-products of plant size and regional variables and of sector (high- or low-tech) and regional variables. The results are shown in Table 11. The table displays the value of the interaction terms as well as the pure coefficients of the regional variables in the probit regressions. Interestingly, what can be seen is that the larger the plant, the lower is the influence of regional population, GDP, GDP growth, labor costs, taxes, and structural funding on the relocation choices. For low-tech plants, none of the interaction terms were statistically significant at conventional levels; this was also true across the groups of both all and small and medium-sized plants in high-tech industries. However, for large, high-tech plants, a positive and significant interaction term was found for the factors of regional population, GDP, labor costs, taxes, accessibility, and structural funding. Thus, one can interpret that for large, high-tech plants the influence on relocation choices stemming from the population in a prospective region, GDP, labor costs, the tax base, and structural funding in that region is higher.

**Table 11:** Robustness analyses, plant-level effects

	(1)	(2)	(3)	(4)	(5)	(6)	(7)	(8)	
	All plants		Small and medium-sized plants		Large plants		Large plants		Baseline variables
	Interaction term	Pure coefficient	Interaction term	Pure coefficient	Interaction term	Pure coefficient	Interaction term	Pure coefficient	
Interaction terms with:									
Plant size							High-tech		
and									
Plant agglomeration	0.0144***	− 0.0946	0.0096	− 0.0507	0.0659**	− 0.6253	− 0.6926***	0.2485**	Yes
	(0.0048)	(0.1489)	(0.0065)	(0.1587)	(0.0319)	(0.2524)	(0.1082)	(0.111)	
Population	− 0.0066***	− 0.0035	− 0.0043	− 0.0388	− 0.0302**	0.6089*	0.3536***	0.0138**	Yes
	(0.0022)	(0.0597)	(0.0029)	(0.06)	(0.0140)	(0.3647)	(0.0580)	(0.2664)	
GDP	− 0.0081***	− 0.3691	− 0.0054	− 0.3176	− 0.0365**	− 1.2427*	0.4298***	− 2.9209***	Yes
	(0.0026)	(0.251)	(0.0035)	(0.2612)	(0.0179)	(0.6379)	(0.0703)	(1.1173)	
GDP growth	− 0.0187***	0.1041***	− 0.0135	0.0656	− 0.0958***	0.7646**	0.1335	− 0.1253***	Yes
	(0.0055)	(0.04)	(0.0086)	(0.0553)	(0.0368)	(0.332)	(0.0887)	(0.0241)	
Labor costs	− 0.0101***	0.9846***	− 0.0066	0.9316***	− 0.0465**	1.8822***	0.5235***	1.382*	Yes
	(0.0033)	(0.3294)	(0.0044)	(0.3525)	(0.0228)	(0.5103)	(0.0855)	(1.0828)	
Taxes	− 0.0134***	0.1195	− 0.0089	0.0556	− 0.0608**	1.0738*	0.6593***	0.7227	Yes
	(0.0043)	(0.1314)	(0.0058)	(0.1363)	(0.0302)	(0.6418)	(0.0925)	(0.6627)	
Accessibility	− 0.0193***	− 0.2001	− 0.0136	− 0.3614**	− 0.085**	2.7149***	0.8663***	0.9396	Yes
	(0.0062)	(0.1706)	(0.0084)	(0.1801)	(0.04)	(0.8935)	(0.1373)	(0.6407)	
Structural funding	− 0.0238*	0.1386*	− 0.0245*	0.1404	− 0.0583***	0.3974***	0.3796***	− 0.4022***	Yes
	(0.0125)	(0.0777)	(0.0141)	(0.0858)	(0.0073)	(0.0605)	(0.0993)	(0.1143)	

This Table displays estimates for the relocation choice of German manufacturing plants. The dependent variable models the relocation decision $Y=1$ or the decision to stay $Y=0$ for each plant. Data are taken from the German Federal Statistical Office and the Statistical Offices of the Federal States, from the Regional Database GENESIS and from INKAR/BBSR. Robust standard errors were computed and are shown in parentheses. ***Denotes significance at the 1% level, **denotes significance at the 5% level, *denotes significance at the 10% level

*Further control variables*. As a final robustness check, further variables were added to the benchmark regression framework and analyses were conducted as to whether they bear additional explanatory power for the relocation choices of plants. Agglomeration diseconomies were tested for by adding a squared term of plant agglomeration. Agglomeration diseconomies could model various negative effects, such as congestion or pollution resulting from a higher degree of agglomeration, which would count against relocation. Moreover, a measure of the relative share of students in the population was considered: this figure may serve as a measure of human capital. However, previous literature has found ambiguous effects of this factor on firm location choices (see, e.g., Arauzo-Carod 2013; Arauzo-Carod and Viladecans-Marsal 2009). The results from the analyses are shown in Table 12. All of the results are statistically insignificant at conventional levels. In terms of economic significance, though, the effects suggest for the case of small and medium-sized plants that diseconomies may play a role, given that the coefficient for these is negative, and the effect of the student share is positive. Those effects are reversed for large plants.

**Table 12:** Robustness analyses, Further explanatory factors

	(1)	(2)	(3)	(4)	(5)	(6)
	All plants		Small and medium-sized plants		Large plants	
	Probit coefficient	*Average marginal effect*	Probit coefficient	*Average marginal effect*	Probit coefficient	*Average marginal effect*
Agglomeration squared	− 0.0228	− 0.0001	− 0.0328	− 0.0002	0.1603	0.0004
	(0.0293)	(0.0002)	(0.0291)	(0.0002)	(0.1374)	(0.0004)
Baseline variables	Yes	Yes	Yes	Yes	Yes	Yes
Wald $\chi ^{2}$	23.39		25.41		188.55	
Prob > $\chi ^{2}$	0.0029		0.0013		0.0000	
Log pseudolikelihood	− 524.289		− 481.211		− 35.953	
Number of plants	43,278		37,639		5639	
Share of students	0.0044	0.00002	0.005	0.00003	− 0.0117	− 0.00003
	(0.009)	(0.00005)	(0.0092)	(0.0001)	(0.0183)	(0.0001)
Baseline variables	Yes	Yes	Yes	Yes	Yes	Yes
Wald $\chi ^{2}$	23.16		24.98		185.39	
Prob > $\chi ^{2}$	0.006		0.003		0.0000	
Log pseudolikelihood	− 524.14		− 481.032		− 35.934	
Number of plants	43,278		37,639		5639	

This Table displays estimates for the relocation choice of German manufacturing plants. The dependent variable models the relocation decision $Y=1$ or the decision to stay $Y=0$ for each plant. Data are taken from the German Federal Statistical Office and the Statistical Offices of the Federal States, from the Regional Database GENESIS and from INKAR/BBSR. Robust standard errors are shown in parentheses for Columns (1), (3), and (5). Standard errors based on the Delta-method were computed and are shown in parentheses for Columns (2), (4), and (6). ***Denotes significance at the 1% level, **denotes significance at the 5% level, *denotes significance at the 10% level

## Conclusion

The present work investigates the relocation decisions of manufacturing plants across the NUTS-3 regions of Germany. It complements previous analyses that focus on new firm activity as well as studies that focus on multiple alternative choices in location decisions by asking the question, “Do plants decide to relocate: yes or no?”, or more simply “Stay or Move?”. It exploits for the first time the rich information provided by the official German regional statistics as well as the official German firm statistics that are maintained by the German Federal Statistical Office and the Statistical Offices of the Federal States. Based on these data, a novel and comprehensive dataset was constructed to investigate the relocation decisions made by German manufacturing plants.

The results reveal that relocation decisions are driven by greater accessibility of regions, as measured by travel times by road and, as such, by the quality of regional road infrastructure. An important relationship is found between the improvement of the accessibility of a region as represented by the regional road infrastructure and travel time and the relocation choice of a plant. Specifically, a 10% decrease in travel time to the three nearest agglomeration centers leads to an increase in relocation probability of approximately 9.5% on average across all plants and 12.5% on average for small and medium-sized plants. This effect is relevant for small and medium-sized plants but not for large plants. A lower travel time makes small and medium-sized plants more likely to move toward another region. Further, the quality of the workforce, as indicated by worker remuneration, positively affects the relocation choice. Again, this effect is relevant for small and medium-sized plants but not for large plants. Moreover, the potential to sell and find capacity in a market appears to be another driving factor for the relocation decision, and this effect appears to be important for large plants.

Through the use of the present regression framework, the computation and interpretation of the effects of these variables in terms of relocation probabilities were possible. The effects appear to be economically and statistically significant. However, a limitation of this research is that the dataset does not provide any manager- or owner-related individual-level variables. Addressing the role of those factors in relocation decisions would be an interesting avenue for future research.

In terms of policy implications, the results of this study provide evidence that policies that endeavor to support firm activities at a regional level need to address regional road infrastructure. Improving road infrastructure and making regions attractive to highly skilled workers appear to be crucial elements that lead to plants deciding to leave the region in which they are located and move to a new region.

## Data Availability

The plant data are taken from the German Federal Statistical Office and the Statistical Offices of the Federal States. The data are confidential but not exclusive and come at a cost, further information can be found at www.forschungsdatenzentrum.de/en/terms-use. The regional INKAR and GENESIS data are publicly available and can be downloaded from the websites of the German Federal Statistical Office and from the BBSR.

## Footnotes

1 See https://www.destatis.de/Europa/EN/Methods/Classifications/OverviewClassification_NUTS.html. The term “district-free city” refers to the administrative status of a city: it does not belong to a district but conducts its own administration. Berlin, for example, is a district-free city. Both districts and district-free cities make up the 401 NUTS-3 regions in the German economy.

2 A different question is that of which factors induce plants to move out of a region; see, e.g., Weterings (2014).

3 In addition, firms pay either—as a variable tax—a corporate tax (*Koerperschaftssteuer*) of 15% or an income tax on profits, depending on the firm’s structure. Firms also pay the so-called solidarity tax (*Solidaritaetszuschlag*) of 5.5%. Data on these items were not available for use in the regressions.

4 An agglomeration center is defined in German regional policy as follows: The population density of the district or district-free city must exceed a limit, calculated as the average of the population density in Germany (523 inhabitants per square kilometer) plus 1.5 times its standard deviation.

5 Many regions in Germany do not receive any funding from the GRW. GRW funding was particularly designed to level inequalities across regions, e.g., across East German regions, or between regions across the former East-West German border.

6 Note that the number of relocating plants could in effect be higher because the data allow for investigation of plants with more than 20 employees. This leaves open the possibility that from 2012 to 2013, a plant could have seen a significant increase or decrease in employment, bringing it above or below that 20-employee threshold and causing it to drop out of the sample, which considers plants with data available for both 2012 and 2013.

7 The firm data carry indicators of the federal state (*Bundesland*) and the community level (LAU2, formerly NUTS-5) in the form of the state and community identifier (*Amtlicher Gemeindeschluessel*). Given the data availability from the regional databases, the plant-level information had to be aggregated and regional identifiers at the NUTS-3 level be constructed.

8 The exception is GDP growth, which shows the effect associated with a 1% increase since the variable is measured in percent.

9 Checks for variance inflation factors were run. No multicollinearity issues were found, the factors were all well below 10.

10 The descriptive statistics, as well as the predicted margins graphics for the sample of small and medium-sized plants and the sample of large plants, are not shown here but are available from the author upon request.

11 The results for large plants cannot be displayed due to a low number of observations and the regulations guaranteeing the anonymity of firms as mandated by German law and the German Statistical Office. Moreover, the regressions that used regional fixed effects did not converge.

## Code availability

The code that was programmed for the analyses is available upon request.
